# Supplementary material for: Aptamer-Modified Erythrocyte Membrane-Coated pH-Sensitive Nanoparticles for c-Met-Targeted Therapy of Glioblastoma Multiforme
Source: Membranes (Basel). 2022 Jul 29;12(8):744. doi: 10.3390/membranes12080744 (PMC9415068; doi:10.3390/membranes12080744)
Supplement: Supplementary file 1 [file membranes-12-00744-s001.zip › membranes-1778286-supplementary.pdf]

Supplementary files

Aptamer-modified erythrocyte membrane-coated pH-sensitive nanoparticles for c-Met-targeted therapy of glioblastoma multiforme

Xianping Liu, Yixin Chen, Daoying Geng, Haichun Li, Ting Jiang, Zimiao Luo, Jianhong Wang, Zhiqing Pang and Jun Zhang

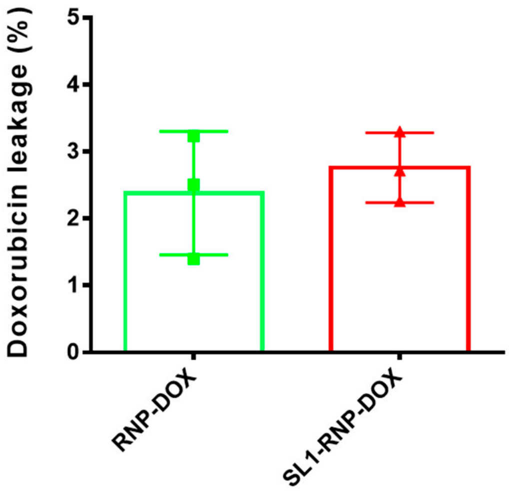

Figure S1 DOX leakage from RNP-DOX and SL1-RNP-DOX after a week storage at 4 ℃ in PBS.

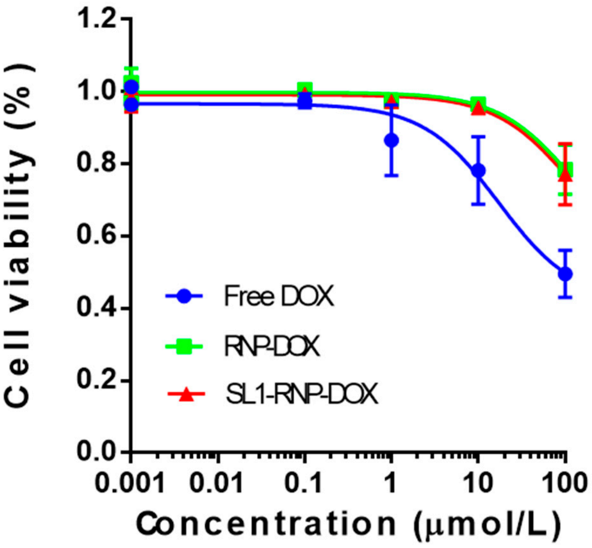

Figure S2 Cell viability of HUVECs treated with free DOX, RNP−DOX, and SL1−RNP−DOX (n = 4).
